# Endocrinological features and epileptic encephalopathy in COX deficiency due to *SCO1* mutations: case series and review of literature

**DOI:** 10.1530/EC-24-0221

**Published:** 2024-09-28

**Authors:** Alessandro Barbato, Giulia Gori, Michele Sacchini, Francesca Pochiero, Sara Bargiacchi, Giovanna Traficante, Viviana Palazzo, Lucia Tiberi, Claudia Bianchini, Davide Mei, Elena Parrini, Tiziana Pisano, Elena Procopio, Renzo Guerrini, Angela Peron, Stefano Stagi

**Affiliations:** 1 Auxo-endocrinology Unit, Meyer Children's Hospital IRCCS, Florence, Italy; 2 Department of Health Sciences, University of Florence, Florence, Italy; 3 Medical Genetics Unit, Meyer Children’s Hospital IRCCS, Florence, Italy; 4 Metabolic and Muscular Unit, Meyer Children's Hospital IRCCS, Florence, Italy; 5 Pediatric Neurology and Neurogenetics Unit and Laboratories, Meyer Children’s Hospital IRCCS, Florence, Italy; 6 NEUROFARBA Department, University of Florence, Florence, Italy; 7 Department of Clinical and Experimental Biomedical Sciences “Mario Serio”, University of Florence, Florence, Italy

**Keywords:** COX deficiency, developmental epileptic encephalopathy phenotypes, epilepsy, growth, hypopituitarism, pathogenic variant, SCO1 gene, short stature

## Abstract

**Context:**

Cytochrome C oxidase (COX) is the fourth component of the respiratory chain and is located within the internal membrane of mitochondria. COX deficiency causes an inherited mitochondrial disease with significant genetic and phenotypic heterogeneity. Four clinical subtypes have been identified, each with distinct phenotypes and genetic variants. Mitochondrial complex IV deficiency nuclear type 4 (MC4DN4) is a form of COX deficiency associated with pathogenic variants in the *SCO1* gene.

**Case description:**

We describe three patients with MC4DN4 with developmental and epileptic encephalopathy (DEE), hypopituitarism, and *SCO1* pathogenic variants. These patients’ phenotypes considerably differ from previously reported MC4DN4 phenotypes as they associate DEE with progressive hypopituitarism and survival beyond the first months after birth. Pituitary deficiency in these patients progressively worsened and mainly involved growth hormone secretion and thyroid function.

**Conclusions:**

Our findings expand knowledge of phenotypic variability in MC4DN4 and suggest that SCO1 is a candidate gene for genetic hypopituitarism and DEE.

**Significance statement:**

Our paper describes three patients affected by MC4DN4 with hypopituitarism and developmental and epileptic encephalopathy (DEE), two features that have never been associated with this condition. In addition, we reviewed the clinical features of all previous cases of MC4DN4 to give the other clinicians a wide picture of the clinical phenotype of this genetic disease. We hope that the publication of our data may help others to identify this disease and consider the chance to analyze the *SCO1* gene in cases of DEE associated with pituitary dysfunction. Our article contributes to expanding the spectrum of genetic hypopituitarism and proposes a model to explain an association between this condition, mitochondrial anomalies, and neurodevelopmental defects.

## Introduction

The term cytochrome C oxidase (COX) deficiency refers to a spectrum of diseases rather than a specific clinical or genetic variant. It includes all clinical conditions associated with a disruption of biogenesis or function of the fourth complex of the mitochondrial respiratory chain. The most used classification of COX deficiencies is based on phenotypic features and includes four subtypes: Leigh syndrome, French-Canadian type, benign infantile mitochondrial variant, and severe infantile mitochondrial type. In addition, a wide spectrum of additional clinical variants not belonging to the former subtypes has been described (1, 2). Mitochondrial complex IV deficiency nuclear type 4 (MC4DN4, OMIM #619048) is an autosomal recessive disorder caused by homozygous or compound heterozygous pathogenic variants in the *SCO1* gene (chromosome 17p13.1), which encodes for an assembly factor of COX (3) Associated phenotypes include severe encephalopathy, hepatopathy, lactate acidosis, and hypertrophic cardiomyopathy. All six previously reported individuals had an early age of onset with demise within the first months of life, except for one patient (4, 5, 6, 7, 8).

This type of COX deficiency was first described by Valnot *et al*.(4) in two patients with metabolic acidosis, hepatomegaly, and neurologic impairment, of whom the first died 5 days after birth and the second at 2 months. Panlobular steatosis and lipidic microvesicular vacuolization were found in liver biopsy; muscle biopsy showed lipid droplets. *SCO1* sequencing identified a compound heterozygosity for a frameshift mutation (c.364_364+1delAG) – which alters the splicing mechanism – and a missense mutation (c.520C>T;p.Pro147Leu) in the copper-binding region CX3C of *SCO1*(4).

A further patient with MC4DN4, a girl described by Stiburek *et al.*(5) had left ventricular hypertrophy and a histological finding of an increased number of mitochondria, intrauterine growth restriction (IUGR), liver enlargement, hypotonia, brain atrophy, and metabolic acidosis. The girl died at 6 months of age due to cardiac failure; genetic analysis showed a homozygous *SCO1* mutation (c.394G>A;p.Gly132Ser) (5).

Leary *et al.*(6) identified a patient with COX deficiency in whom a fatal encephalopathy and lactic acidosis were caused by compound heterozygous mutations in *SCO1* (c.880A>G; p.Met294Val);(c.277_279delGTTinsTAA;p.Val93*). The patient died at 5 months of age.

Brix *et al.*(7) described a girl born preterm due to severe IUGR, who developed severe lactic acidosis and died at age 1 month. An early genetic diagnosis identified a homozygous in-frame deletion (c.317_319delGAG;p.Gly106del) in *SCO.* Kose *et al.*(8) described a 6-year-old child with increased lactate, basal ganglia involvement, cardiomyopathy, and hearing loss carrying the homozygous variant (c.715A>G;p.Arg239Gly), which was classified as of unknown significance (VUS).

Kurian *et al.*(9) described neonatal onset refractory developmental epileptic encephalopathy (DEE) in one patient, with the absence of terminal phalanges and liver enlargement without steatosis. *SCO1* sequencing was negative, but liver biopsy identified complex IV deficiency. We describe two siblings and a third unrelated patient with progressive panhypopituitarism and DEE in whom we identified homozygous missense *SCO1* mutations. To our knowledge, DEE and pituitary involvement have not yet been associated with MC4DN4.

## Subjects and methods

We conducted a retrospective review of patients with MC4DN4 diagnosed and followed up at Meyer Children’s Hospital IRCCS to characterize their phenotypes. In all patients, whole exome sequencing (WES) was performed. All three patients were born to non-consanguineous healthy parents and had no previous family history of genetic diseases. Clinical and imaging features of our patients are summarized in Table 1; the main clinical and genetic features of our and previously reported patients are summarized in Table 2. This study was performed in accordance with the principles of the Declaration of Helsinki. Informed consent for genetic testing and clinical description was collected from the parents. The study was approved by the local Pediatric Ethical Committee of Tuscany.

**Table 1:** Summary of clinical and radiological features of the three patients.

Case	Height (SDS)	BMI (SDS)	Height (SDS) follow up	BMI (SDS) follow up	Endocrinological features	Seizure type (ILAE 2017) and onset	EEG	Anti-epilectic medications	Brain MRI	Hepatic US
Patient 1 (M)	−5.34	0.57	−4.90	1.41	GHD, CH, AI, CHG	Generalized clonic 2 years	FT high amplitude spike and slow wave complexes(sleep)	Valproate acid, levetiracetam, topiramate, clobazam	Ventriculomegaly. Hypotrophy of the cerebellar hemispheres and of the vermis.	Steatosis
Patient 2 (F)	−3.16	2.33	−2.06	2.31	GHD, CH	Generalized tonic-clonic 2 years	Slow background activity at 5–6 Hz, bursts of large angular slow waves (awake)	Phenobarbital, clobazam, lacosamide	Mildly reduced white matter volume, a thin corpus callosum, ventriculomegaly. Hypotrophy of the cerebellar hemispheres and of the vermis.	N.A.
Patient 3 (M)	−2.94	0.48	−5.1	0.03	GHD, CH, AI, CHG	Generalized motor seizures 22 years	N.A.	Valproate acid	T2 hyperintensity of the bulbar olives. Atrophy of the cerebellar hemispheres and of the vermis.	Steatosis

AI, adrenal insufficiency; CH, central hypothyroidism; CHG, central hypogonadism; FT, frontotemporal; GHD, growth hormone deficiency; N.A., not assessed; SDS, standard deviations; US, ultrasound.

**Table 2:** Clinical features and genetic variants of SCO1 gene in all previously described cases of MC4DN4.

Case reference		Age	Clinical features	*SCO1* variants	ACMG classifications
1	Valnot *et al.*, 2000	2 months (death)	Hypotonia, metabolic acidosis, hepatic disfunction, hypoglicemia, bradicardia	(c.364_364+1delAG);(c.520C>T) (p.?);(p.Pro147Leu)	P (PVS1, PM2, PP5); LP (PP3, PM2, PM5)
2	Valnot *et al.*, 2000	5 days (death)	Metabolic acidosis, encephalopathy	(c.364_364+1delAG);(c.520C>T) (p.?);(p.Pro147Leu)	P (PVS1, PM2, PP5); LP (PP3, PM2, PM5)
3	Stiburek *et al.*, 2009	6 months (death)	IUGR, hypotonia, hepatopathy, hypertrophic cardiomyopathy, encephalopathy, lactate acidosis	(c.394G>A);(c.394G>A) (p.Gly132Ser);(p.Gly132Ser)	VUS (PM2, PP3, PP5)
4	Leary *et al.*, 2013	5 months (death)	Encephalopathy, lactate acidosis	(c.880A>G);(c.277_279delGTTinsTAA) (p.Met294Val);(p.Val93*)	VUS (PM2); LP (PVS1, PM2)
5	Brix *et al.*, 2019	1 month (death)	IUGR, hypoglicemia, severe lactate acidosis	(c.317_319delGAG);(c.317_319del GAG) (p.Gly106del);(p.Gly106del)	VUS (PM2,PM4)
6	Kose *et al.*, 2021	6 years (2021)	Lactate acidosis, encephalopathy, cardiomiopathy, hypotonia, hearing loss	(c.715A>G);(c.715A>G) (p.Arg239Gly);(p.Arg239Gly)	VUS (PM2)
7	Patient 1, our case	15 years (2024)	Psychomotor delay, DEE, hypopituitarism, hepatic steatosis, mild aortic insufficiency	(c.685G>A);(c.685G>A) (p.Gly229Ser);(p.Gly229Ser)	P (PM2, PP1, PP3, PP4)
8	Patient 2, our case	10 years (2024)	Psychomotor delay, DEE, hypopituitarism	(c.685G>A);(c.685G>A) (p.Gly229Ser);(p.Gly229Ser)	P (PM2, PP3, PP1, PP4)
9	Patient 3, our case	35 years (2024)	Psychomotor delay, hypotonia, epilepsy, hypopituitarism, hepatic steatosis	(c.424C>G);(c.4242C>G) (p.Leu142Val);(p.Leu142Val)	LP (PM2, PP3, PP1, PP4)

DEE, developmental and epileptic encephalopathy; IUGR, intra-uterine growth restriction; LP, likely pathogenetic variant; P, pathogenetic variant; VUS, variant of unknown significance.

### Patient 1, male, 15 years old

**Figure 1:**
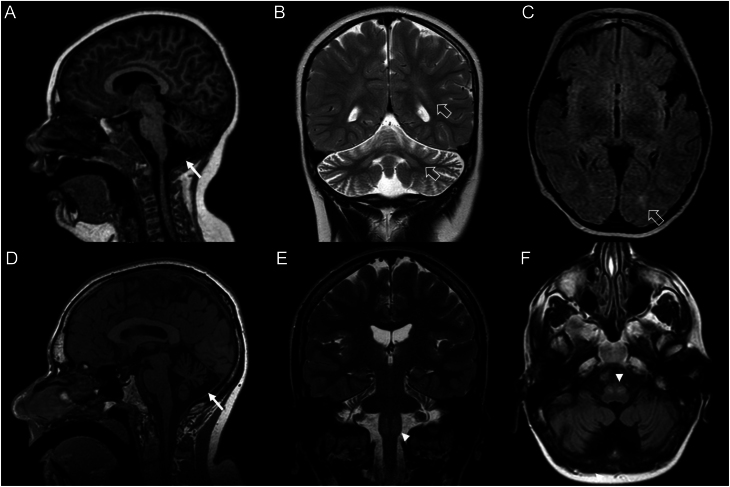
Brain MRI images: A, B, C patient n1, D, E, F patient n2. (A, D) sagittal T1-weighted image; (B, E) coronal T2-weighted image; (C, F) axial FLAIR T2-weighted image. The study shows cerebellar atrophy (white arrow), cerebral atrophy with widening of the Sylvian fissures, bilateral superior olivary nuclei increased signal on FLAIR and T2-weighted images (arrowhead), and posterior parietal and cerebellar white matter hyperintensity on FLAIR and T2-weighted images (empty arrow).

Prenatal history was unremarkable. Early neurologic development was normal until 7 months. Subsequent milestones were delayed, and signs of loss of acquired skills ensued. Brain magnetic resonance imaging (MRI) at 18 months showed mild white matter loss, thin corpus callosum, ventriculomegaly, and dilated fronto-parietal subarachnoid spaces (Fig. 1).

At age 2, he exhibited severe developmental delay and epilepsy with generalized tonic or clonic seizures worsened by fever or infections. EEG recordings, during sleep and wakefulness, revealed high-amplitude spike and slow wave complexes over the frontotemporal regions. Anti-seizure treatment with different drugs did not control the seizures.

At follow-up brain MRI at 6 years and 2 months of age, initial abnormalities and signs of progressive cerebellar atrophy had worsened. An abdominal ultrasound revealed liver enlargement with steatosis. Metabolic assessment repeated over the years showed normal ranges for plasmatic amino acid profile, alanine, organic acid urinary excretion, oxidoreductive cellular balance, the lactate/pyruvate ratio, and plasmatic sialotransferrin profile. Ammonia levels and hemogasanalysis remained normal over the years. Muscle biopsy showed mild COX and succinate dehydrogenase (SDH) reduction in histochemical analysis despite no impairment being detected in mitochondrial respiratory chain activity.

At 9 years of age, the child exhibited DEE with spastic quadriplegia and developed deterioration of thyroid function with a progressive decrease in free tetra-iodothyronine (FT_4_) and a mild increase in thyroid-stimulating hormone (TSH) levels. Based on these findings, a first endocrinological assessment was performed. The child was prepubescent and, as he was unable to stand, length was measured. According to growth charts for Italian boys older than 12 months, severe growth delay was present, with height at −5.34 s.d. Weight was within the normal range at 0.57 s.d. Laboratory tests for thyroid-specific autoimmunity were normal and thyroid ultrasound was unrevealing. Substitution therapy with levo-thyroxine (L-T_4_) was started(10). Delayed bone age based on Greulich and Pyle was observed at wrist X-ray. A growth hormone (GH) stimulation test with arginine was scheduled but not promptly performed since recurrent respiratory infections prompted frequent hospitalizations. The patient rapidly improved when treated with endovenous corticosteroids. Based on clinical features and laboratory findings of low serum cortisol and ACTH levels, secondary adrenal insufficiency was diagnosed. A tetracosactide stimulation test did not highlight a poor secretory response, a feature coherent with recent onset secondary corticoid deficiency(11). Substitution therapy with hydrocortisone was started.

Arginine stimulation test and L-3,4-dihydroxyphenylalanine (L-DOPA) stimulation test were performed to measure GH secretion. In both tests, there was a deficient response based on consensus guidelines(12). Moreover, gonadotropin titers and testosterone were persistently reduced. A diagnosis of panhypopituitarism was made, and synthetic GH analog was started at a dose of 0.2 mg/kg per week.

Since previous genetic tests (karyotype, array-CGH, and *TSEN54* sequencing) were unrevealing, WES was performed on the child and his mother, father, and sister. A homozygous variant in *SCO1* (c.685 G>A; p.Gly229Ser) was identified in the proband and his sister (patient 2); their parents were heterozygous carriers. The variant was absent from gnomAD v4 and was not reported in the literature. These molecular findings allowed a diagnosis of MC4DN4.

At the age of 14, the boy was still prepubescent. Therefore, a gonadotropin-releasing hormone (GnRH) stimulation test was performed, revealing reduced peak levels of luteinizing hormone (LH). Topical testosterone was started to induce puberty(13).

Cardiological assessment revealed first-degree atrioventricular block, septal hypokinesis with preserved ejection fraction, and mild aortic insufficiency. The patient is now in follow-up to assess appropriate progression of puberty and response to substitution therapies.

### Patient 2, girl, 10 years old

Patient 2 was the younger sister of patient 1. Her pre-perinatal history was unremarkable. After an early normal development, axial hypotonia and language delay were noticed from the seventh month of life. At the age of 2, clonic seizures and a transient increase of lactates became apparent. Complete metabolic assessments were unrevealing. Brain MRI showed mildly reduced white matter volume, a thin corpus callosum, and signal abnormalities on both dentate nuclei (Fig. 1). Electroneurography was consistent with peripheral motor neuropathy, which was clinically associated with spastic diplegia and a DEE. Her EEG showed irregular background activity at 5–6 Hz with bursts of large angular slow waves, predominantly over the anterior regions. Anti-seizure medications were introduced, including phenobarbital, clobazam, and lacosamide.

A first endocrinological evaluation was performed at 5 years of age as the girl had low FT_4_ levels and in-range TSH, disclosing a central hypothyroidism. The girl was prepubescent. Replacement therapy with L-T_4_ was started, and she underwent periodic follow-up. According to Cacciari *et al.* growth charts for Italian girls, the girl showed a height at −3.16 s.d. After genetic testing was performed on her brother and he developed panhypopituitarism, arginine, and combined arginine+sermorelin stimulation tests were performed, showing a deficient response to stimulation. Therefore, a synthetic GH analog was introduced at 9 years. The tetracosactide stimulation test was normal.

The homozygous variant in *SCO1* (c.685C>A;p.Gly229Ser) was found. Cardiological assessment with ECG and echocardiogram was unrevealing. The patient is in follow-up to monitor her response to growth hormone therapy and identify any potential involvement of other components of the pituitary axis.

### Patient 3, male, 35 years old

Born at term from a first pregnancy, the patient developed difficulties in feeding after birth. Neuromotor delay was noticed at 4 months of life. During the first years of life, quadriparesis with more severe involvement of lower limbs was noticed. Language skills did not emerge. A skin biopsy supported a clinical diagnosis of cutis laxa. Repeated brain MRIs revealed progressive cerebellar atrophy, bulging of bulbar olives, periventricular white matter hyperintensity, and a shrunken adenohypophysis with empty sella (Fig. 1). These findings remained stable, except for the progression of cerebellar atrophy, on repeat MRIs up to age 30 years.

At 12 months, growth delay was observed, but GH stimulation tests were only performed at the age of 14 years due to irregular follow-up. A deficient response was observed to stimulation tests with clonidine and arginine, and GH substitution therapy was started. In addition, laboratory tests showed low TSH, FT_4_, ACTH, and serum cortisol. Gonadotropin levels were persistently reduced, without any sign of puberty. Substitution therapy with L-T_4_, testosterone, hydrocortisone, and then cortisone acetate was started.

Generalized tonic-clonic seizures triggered by intermittent light stimulation appeared at 22 years, after which valproate treatment was started. Metabolic assessments performed over the years indicated impaired energy metabolism, with high levels of alanine and lysinemia in the post-prandial plasmatic amino acid profile. Organic acid urinary excretion was normal, as was the oxidoreductive cellular balance with a normal lactate/pyruvate ratio; however, mild paradoxical ketosis was present. Karyotype and SNP+CGH array were normal. Exome sequencing showed a homozygous variant (c.424C>G;p.Leu142Val) in the *SCO1* gene; both parents were carriers of the same variant. The variant, not reported in literature, was reported once, at a heterozygous state, in the gnomAD v4 database (1 out of 628,782 alleles; 0 homozygous). Cardiologic assessment showed normal cardiac function; abdominal ultrasound detected hepatic steatosis with normal liver enzymes.

## Genetics and molecular biology

**Figure 2:**
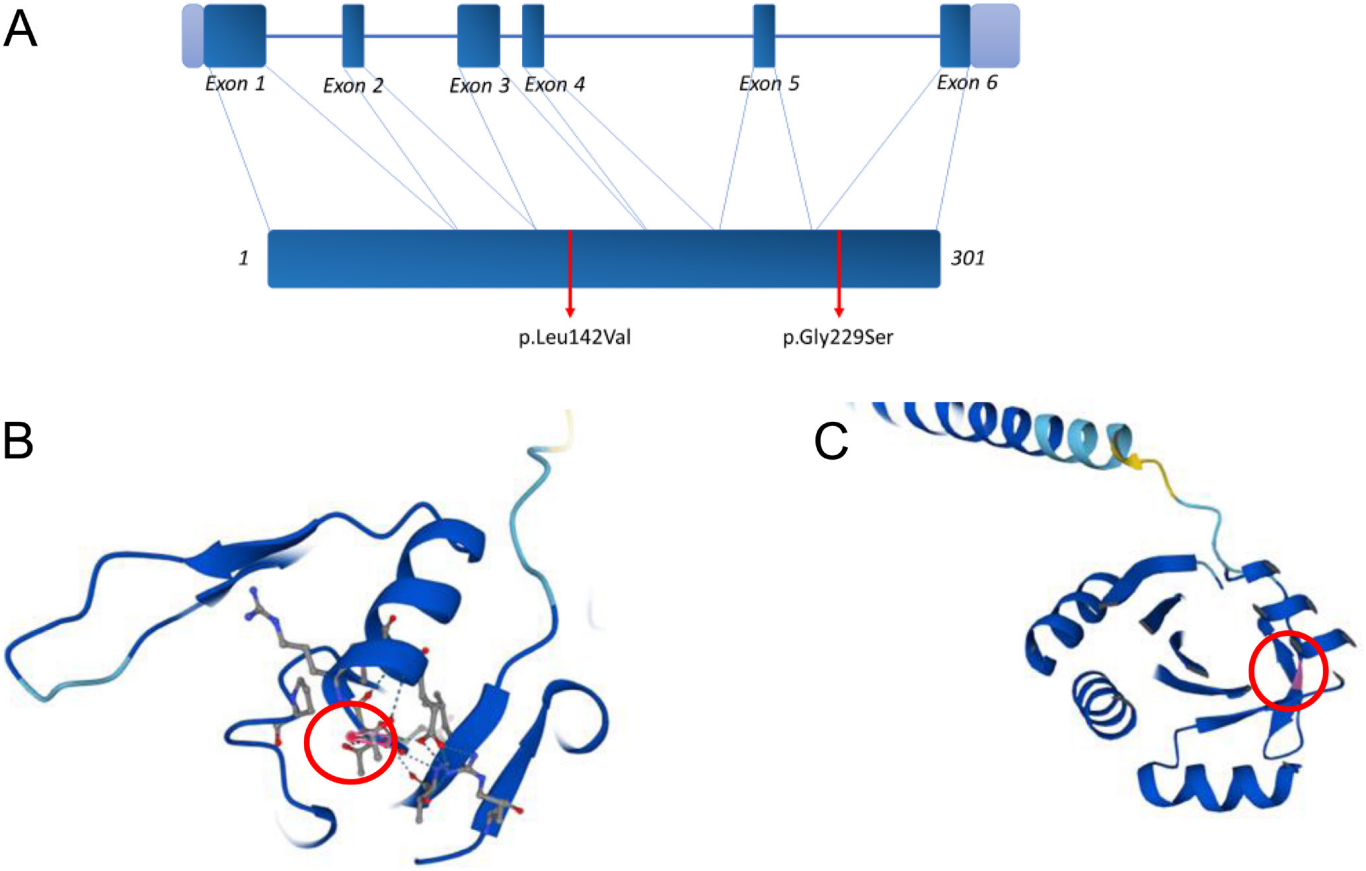
(A) Location on SCO1 gene of the two identified mutations modified from Valnot *et al.*; (B) 3D model of SCO1 protein with mutation site c.685 G>A (p.Gly229Ser); (C) 3D model of SCO1 protein with mutation site c.424C>G (p.Leu142Val). 3D models were realized with AlphaFold on www.uniprot.org.

COX is a multimeric complex located in the internal mitochondrial membrane and includes 13 subunits encoded by both mitochondrial and nuclear genes. It acts as a catalyzer for the transfer of electrons from C cytochrome to molecular oxygen (1). *SCO1* is a copper-binding chaperone encoded by the *SCO1* nuclear gene, located in position 17p13.1, which includes six exons (14). The protein is involved in the delivery of copper to the binuclear copper site of the MT-CO2 subunit, along with protein *SCO2* (3, 15). MT-CO2 is one of the core components involved in COX biogenesis and part of the highly preserved catalytic core of COX (1, 3). The role of *SCO1* in COX biogenesis was shown to be related to the stability of this multimeric complex rather than its expression (15). Indeed, in an experimental cellular model of *SCO1* deficiency, Leary *et al.* observed a reduction of fully assembled COX without a reduction of assembly intermediates of MT-CO2 (15). The c.685 G>A variant of the *SCO1* gene leads to the amino acid substitution Gly229Ser. This molecular alteration involves a peptidic sequence between the β-strand 225-228 and the α-helix 231-239 (Fig. 2) and interferes with folding processes. Folding stability analysis computed using the PoPMuSiC-2.0 (16) and Site Directed Mutator (SDM) (17) tools, suggests the variant has a destabilizing impact (∆∆G in kcal/mol: −1.91 for PopMuSiC and −1.04 for SDM). According to ACMG (American College of Medical Genetics and Genomics) guidelines (18) and the SVI ClinGen recommendations (19), the variant c.685G>A is a pathogenic variant as it meets the PM2 supporting, PP3 strong, PP1 supporting, and PP4 strong criteria. The c.424C>G (p.Leu142Val) variant is situated in the β-strand 141–144 (Fig. 2) and could be involved in maintaining the tridimensional conformation of the protein. Folding stability analysis computed using the PoPMuSiC-2.0 (16) and Site Directed Mutator (SDM) (17) tools, suggests the variant has a destabilizing impact (∆∆G in kcal/mol: −1.45 for PopMuSiC and −1.41 for SDM). According to ACMG guidelines, the variant c.424C>G is a likely pathogenic variant as it meets the PM2 supporting, PP3 strong, PP1 supporting, and PP4 moderate criteria (18, 20).

*SCO1* gene structure and the molecular structure of the *SCO1* protein with mutation sites are shown in Fig. 2.

## Discussion

Panhypopituitarism associated with COX deficiency and *SCO1* mutations has not been previously described. Animal models of *SCO1* mutations have been subjected to organ-specific gene ablation for the liver or heart (20, 21) and show a growth restriction associated with failure to thrive. The clinical features of the three patients described herein were considerably different from both animal models and previously reported individuals with MC4DN4. Cardiac involvement was absent or mild in our patients, and liver steatosis was not yet associated with hepatic dysfunction. Our patients reached ages that go beyond those reported in the literature, suggesting that survival beyond childhood is possible in MC4DN4. We hypothesize a correlation between variant type and phenotype severity, as missense variants might result in a milder phenotype than truncating variants, thus explaining why our patients, and possibly the one described by Kose *et al.*(8) survived beyond infancy. Almost all previously described affected individuals were compound heterozygous for a truncating and a missense variant or homozygous for two truncating variants and manifested a more severe phenotype leading to premature death. Further functional studies are needed to prove our hypothesis. Pituitary dysfunction in two of the three affected individuals was not associated with significant alterations of hypothalamic and pituitary districts on imaging.

Short stature is commonly associated with disorders of mitochondria, and disorders of the endocrine system are frequently reported in patients with mitochondrial diseases and defects of mitochondrial oxidative phosphorylation (OXPHOS) (23, 24). Therefore, several scenarios should prompt endocrinologists to consider a mitochondrial etiology for endocrine problems that are ‘atypical’ or associated with neurological involvement.

The trend of growth of all our patients highlights a precocious deficit of GH secretion, with a progressive reduction in TSH. Both patient 1 and patient 3 developed secondary adrenal insufficiency. A potential confounding factor in identifying the role of GH deficiency in patients with poor linear growth may be concurrent neuromotor impairment, which may lead clinicians to regard poor mobility as the main cause of reduced growth. Gonadotropin secretion was present in patient 1 and patient 3, with a rapid response to topical testosterone being observed in patient 1. We suggest periodically assessing pituitary function in MC4DN4-affected individuals. The choice of sermorelin combined with arginine to assess GH secretion in the female patient was driven by the need to rule out the involvement of hypothalamic neurons alone, due to the *SCO1* mutation’s effects on neural tissue metabolism (25).

Absence of respiratory chain function abnormalities in muscle biopsy in patients 1 and 2 was in line with observations in other forms of COX deficiency (2). In patient 1, mild reduction of SDH was also detected at histochemical analysis, a finding not explained by *SCO1* genetic variants alone. Nonetheless, the absence of pathogenic variants in genes encoding for components of SDH and the normal activity of this component in the respiratory chain activity assessment led to the exclusion that the reduced levels of SDH could be causative of patient 1’s clinical features.

We propose a biphasic model to understand the different timings of neurologic symptoms and pituitary dysfunction in our patients. Brosel *et al.*(26) highlighted a preferential expression of *SCO1* compared to *SCO2* in the liver and blood vessels in mammals, but this finding does not completely explain the clinical features of MC4DN4. Early neurological features could be related to developmental brain abnormalities due to defective metabolic programming of neural progenitor cells (27). Defective *SCO1* functioning would cause developmental delay and deterioration, seizures, cerebellar atrophy, and an increased susceptibility of hypothalamic and pituitary cells to energetic metabolism defects, leading to progressive deterioration of the hypothalamic–vascular–pituitary axis (27). This model might explain the slow occurrence of pituitary dysfunction. In addition, the alteration of the hypothalamic-pituitary axis might be associated with abnormal interaction between vessels transporting hypothalamic peptides and pituitary cells, as SCO1 is highly expressed in the endothelium (26, 28).

## Conclusions

MC4DN4 is an ultra-rare genetic disorder with heterogeneous clinical features. Here, we have shown that the involvement of the hypothalamic-pituitary axis and DEE may be novel manifestations of the disease. Therefore, we suggest including pituitary function assessment in evaluations following diagnosis of MC4DN4 and considering *SCO1* as a candidate gene in the diagnostic assessment of hypopituitarism and DEE. Further research is needed to better understand the underlying mechanisms and potential therapeutic strategies for individuals with MC4DN4.

## Declaration of interest

The authors declare that there is no conflict of interest that could be perceived as prejudicing the impartiality of the study reported.

## Funding

This study was supported in part by the Funds of the Ministry of Healthhttp://dx.doi.org/10.13039/100009647 for Current Research 2024.

## Author contribution statement

AB carried out the data collection and interpretation and participated in the design of the study. GG carried out the genetic data collection and interpretation. MS carried out the metabolic data collection and interpretation. FP carried out the metabolic data collection and interpretation. SB carried out the genetic data collection and interpretation. GT carried out the genetic data collection and interpretation. VP carried out the genetic data collection and interpretation. LT carried out the genetic data collection and interpretation. CB carried out the genetic data collection and interpretation. DM carried out the genetic data collection and interpretation. EP carried out the genetic data collection and interpretation. TP carried out the neurologic data collection and interpretation. EP carried out the metabolic data collection and interpretation. RG participated in the design and coordination of the study. AP participated in the design and coordination of the study. SS conceived the study and participated in its design and coordination. All authors read and approved the final manuscript.
